# Associations between resting‐state neural connectivity and positive affect in social anxiety disorder

**DOI:** 10.1002/brb3.3006

**Published:** 2023-04-16

**Authors:** Corinne N. Carlton, Ligia Antezana, John A. Richey

**Affiliations:** ^1^ Department of Psychology Virginia Tech Blacksburg Virginia; ^2^ Department of Psychiatry University of Pittsburgh School of Medicine Pittsburgh Pennsylvania

**Keywords:** positive affect, resting‐state fMRI, reward, social anxiety disorder

## Abstract

**Introduction:**

Social anxiety disorder (SAD) has been characterized by deficits in social motivation and lack of reactivity to pleasurable stimuli (i.e., positive affect; [PA]). Recent neuroimaging work has shifted toward examining positively valenced motivational systems in SAD focused on reward responses. However, little is known about the associations of reward connectivity and PA in individuals with SAD. As such, the purpose of the current study was to determine whether connectivity among key units of reward neurocircuitry meaningfully relate to PA and whether these key units are more heterogeneous in SAD as compared to controls.

**Methods:**

Thirty‐one participants who met diagnostic criteria for SAD and 33 control participants were included (*M_age_* = 24.8, *SD* = 6.9; 55% cisgender man). Seed‐based timeseries correlations were conducted in NiTime to extract region of interest (ROI) coupling correlation strength values. ANOVAs were carried out to assess whether individuals with SAD differed in ROI‐to‐ROI connectivity strength as compared to controls. Correlations and variance analyses were also conducted to examine the relationship between ROI‐to‐ROI connectivity strength and PA, as well as heterogeneity in connectivity strength and PA expression.

**Results:**

Weaker connectivity between the left and right orbital frontal cortex was observed when comparing the SAD to the control group. Within the SAD group, PA was associated with several reward‐related ROI couplings; however, these links were not observed among controls. Results further demonstrated that individuals with SAD had significantly more variability in reward connectivity strength as compared to controls.

**Conclusion:**

Overall, these results provide emergent evidence for the association between reward regions and PA in individuals with SAD. Additionally, these findings show that individuals with SAD demonstrate greater heterogeneity in reward connectivity.

## Graphical abstract

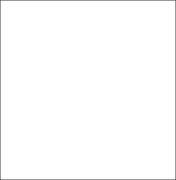

## 1 INTRODUCTION

Social anxiety disorder (SAD) is a common and impairing psychiatric disorder that is characterized by persistent concern or fear of negative evaluation and excessive avoidance of one or more social situations (American Psychiatric Association, 2013). Approximately 12% of adults experience social anxiety symptoms during their lives; ranking SAD as one of the most common psychiatric disorders (Kessler et al., 2005). Prior research has demonstrated that symptoms of SAD can be effectively reduced with the use of cognitive behavioral therapy (CBT), which mainly targets fear‐ and threat‐based systems (Gordon et al., 2014). Despite widespread evidence supporting the utility of CBT in this population, this treatment approach results in only modest rates of diagnostic remission (Heimberg et al., 1998; Loerinc et al., 2015). Collectively, these findings suggest that current frontline treatment approaches do not target critical mechanisms involved in the maintenance of SAD. Consequently, there remains a crucial need to identify and characterize potential mechanisms involved in the maintenance of SAD. One proposed mechanism of maintenance is diminished positive emotionality and associated links to reward functioning. However, this area of research remains in its infancy. As such, the purpose of the current study was to determine if individual differences in positive affect in adults with SAD are concurrently linked to functional connectivity of key cortical and subcortical structures involved in reward processing, as measured by resting‐state functional connectivity.

Neuroimaging approaches have predominantly focused on predictions made by cognitive behavioral theories which focus almost exclusively on negative affect, fear, and threat‐related arousal (e.g., Hattingh et al., 2013; Goldin et al., 2009; Mizzi et al., 2022; Phan et al., 2006; Schmidt et al., 2010). For example, prior work using task‐based fMRI has supported the involvement of brain areas related to fear and threat‐related arousal (e.g., the amygdala; limbic system), such that individuals with SAD demonstrate increased activation of these areas during threat‐related tasks such as viewing harsh faces or socially threatening words (e.g., Goldin et al., 2009; Phan et al., 2006; Schmidt et al., 2010). More recently, there has been an increasing appreciation of work that aims to understand positively valenced motivational systems in psychiatric disorders more broadly (Casey et al., 2014; Insel, 2014). Within positive valence systems, prior neuroimaging work has focused on the neurobiology of reward pathways as they relate to social (e.g., reciprocity, positive social feedback) and nonsocial (e.g., money, food, addictive drugs) reward processing (Kawamichi et al., 2016; Gorka et al., 2018; Wake & Izuma, 2017). Collectively, this work has suggested that the reward network is recruited when presented with incentives, which includes activation of the ventral striatum (VS), orbitofrontal cortex (OFC), and ventromedial prefrontal cortex (vmPFC; Izuma et al., 2008; Rademacher et al., 2010; Rilling et al., 2008; Spreckelmeyer et al., 2009).

Naturalistic processing of social rewards and associated neurofunctional substrates are implicated in normative social functioning and as such, systematic dysfunction within neural networks involved in reward processing could reveal clues regarding the ways in which disorders such as SAD develop and persist. Consequently, improvements in the general understanding of functional connectivity in reward processing units have potential to advance knowledge of the genesis and maintenance of this disorder. The processing of reward is complex but is almost certainly mediated by dopaminergic neurons originating from the ventral tegmental area (VTA), which extend to the VS, OFC, vmPFC, and the anterior cingulate cortex (ACC) forming an integrated mesolimbic dopamine pathway (Haber & Knutson, 2010). Major hubs within the mesolimbic dopamine pathway have specific functional roles in elaborating and sustaining value signals and translating expected value into motivated action (Haber & Knutson, 2010). For example, the VS has been previously implicated in the refinement and control of motor movement; however, more current research has also suggested a role for the VS in decision‐making, and in particular, a role in expected action‐outcome contingencies and reward anticipation (Balleine et al., 2007; FitzGerald et al., 2014; O'Doherty, 2004). Functionally, the VS elicits robust activity while processing dynamic social incentives, reflecting its role in motivation to obtain social rewards and avoid social punishment (e.g., Kohls et al., 2013). Diffuse connections project from the VS to prefrontal cortical regions including the OFC, which in turn has a known role in sustaining expected value for various types of rewards (Kahnt et al., 2012; Kringelbach & Rolls, 2004). The OFC has collectively been implicated as crucial for flexibly modulating appropriate social behaviors in response to positive and negative social cues during active social interactions and changing reward contingencies (Bachevalier et al., 2011; Balleine et al., 2007; Hare et al., 2008; Kahnt et al., 2012).

Other implicated areas of influence on the VS include the vmPFC and the ACC. Functional roles of the vmPFC over the VS include encoding the magnitude of anticipated rewards and integrating cost and benefit information to inform choice (Ballard & Knutson, 2009; Kahnt et al., 2011). The vmPFC has also been shown to be involved in self‐referential processing (Kelley et al., 2002; Mitchell et al., 2006) and acts as a gate for sensory input associated with interpersonal cues during value processing done by the ventral striatum (Sumiya et al., 2017). The ACC has been implicated as a primary processing area for retaining history of encountered rewards, computation of reward trajectories, and the transformation of decision variables into choices (Boorman et al., 2013; Wittmann et al., 2016). Collectively, these areas within the mesolimbic reward pathway are integral for anticipating, assessing, and making decisions in socially rewarding situations; making them a potentially profitable target for examining their role as it relates to PA deficits within SAD (Ikemoto & Panksepp, 1999; Saddoris et al 2015; Schultz, 1998; Schultz, 2000). Given the integral role of these areas (i.e., VS, OFC, vmPFC, and ACC), we selected these areas in the present study as theory‐driven ROIs in subsequent analyses.

Regarding social incentives, Richey and colleagues (2017) demonstrated that individuals with SAD experience diminished anticipatory activity in the VS during socially salient contexts (e.g., positive social incentives) that were independent of enhanced activity responses seen in threat and arousal systems (i.e., hyperactivity of amygdala). Additionally, in other work investigating reward circuitry activation in the context of social rewards and punishment, individuals with SAD demonstrated relatively less activation of reward regions during a Social Incentive Delay task (Spreckelmeyer et al., 2009), suggesting that individuals with SAD may have a relative lack of motivational preference for social reward as compared to controls (Cremers et al., 2015). Moreover, Becker and colleagues (2017) reported that individuals with SAD show a lack of VS activation to positive social feedback while under observation. Furthermore, during reciprocity tasks, individuals with SAD show similar striatal responses to a positive reciprocity outcome regardless of the partner's actual reciprocity rates or lack thereof, suggesting a lack of modulation of reward areas when processing social rewards and situational cues (Sripada et al., 2013). Collectively, these results suggest that there are aberrant activation patterns within reward regions in adults with SAD regarding social contexts that may be less impacted by positive social stimuli.

Deficits in PA have been indicated as potential etiologic and maintenance factors that are unique to SAD as compared to other anxiety disorders and cannot be explained by comorbidity with depression (Alden et al., 2008; Brown et al., 1998; Kashdan, 2007; Watson et al., 1988). Moreover, adults with SAD anticipate a lower likelihood of positive events (Gilboa‐Schechtman et al., 2000), and demonstrate a reduced ability to sustain positive emotions and experiences (Eisner et al., 2009). Related work has further identified a tendency to allocate attention away from positive social stimuli in adults with SAD (Taylor et al., 2010). Moreover, adults with SAD similarly report decrements in the enjoyment of and the motivational pursuit of social interactions, and expression of PA (Trew & Alden, 2012). Taken together, these findings suggest that patterns of diminished PA in individuals with SAD may be linked with disruptions of social reward processing that ultimately may negatively influence interpersonal functioning. Thus, understanding the pathways through which diminished PA is maintained in SAD is crucial for informing the development of increasingly targeted clinical therapies for PA deficits among affected individuals. This work provides an initial step in identifying neural mechanisms of diminished PA in SAD, which may be useful in developing targeted interventions (Li et al., 2022; Siegle et al., 2012; White et al., 2022), such as mindfulness‐based and positive affect interventions (Carlton et al., 2020; Craske et al., 2019; Richey et al., 2019; Strege et al., 2018; Taylor et al., 2020).

While it is increasingly appreciated that some, although not all individuals with SAD demonstrate PA deficits, the broad mechanisms that contribute to this heterogeneity remain generally unknown. Prior theoretical and empirical work has suggested that a surprisingly distinctive pathway may lead to alterations in reward neurobiology among adults with SAD based primarily on patterns of reinforcement history (Richey et al., 2019; Spence & Rapee, 2016). Specifically, intersecting biological and cognitive factors, along with adverse life events and peer relationships may influence the emergence of anhedonic symptoms in a subset of adolescents and adults with social anxiety through a specific pathway by which adverse life events amplify the impact of coping outcomes. Adverse life events appear to influence the development of social anxiety symptoms when they are inescapable, uncontrollable and persistent such as family violence (Binelli et al., 2012) critical and cold parenting practices (Norton & Abbott, 2017) and peer victimization (Siegel et al, 2009). It has been specifically theorized elsewhere (Richey et al., 2019) that the persistence of such events over time may lead to anhedonic symptoms among a select few because a subset of socially anxious individuals cease to actively expend effort to cope with adversity, when it is determined through experience that the expenditure of effort is futile (i.e., effort expenditure does not resolve the stressor or its precipitating factors). This process of deactivation amid persistent social adversity is further consistent with previously described pathways for the development of depressive symptoms generally and anhedonia in particular, as predicted by hopelessness models (e.g., Abramson et al., 1989). When considering the putative reinforcement history among this emergently anhedonic subset of socially anxious individuals, it stands to reason that through repeated pairings of effort expenditure and failure to mitigate sources of social stress, that the net result is a narrowly defined form of learned social helplessness. Put another way, the intrinsically motivating properties of social interaction may become uncoupled from the environmental stimuli that preceded them through repeated thwarting of coping efforts. As such, the functional properties of neural circuits that manage reward processes may become semipermanently altered through the principles of experience‐dependent plasticity (e.g., Kelly & Castellanos, 2014). By mapping the effects of this theorized experience‐dependent change at the macro level of large‐scale functional networks, we may be able to better devise and measure intervention concepts that are aimed at restoration of function.

Accordingly, we specifically anticipated that we would be able to observe differences between adults with social anxiety and nonxanxious matched control participants in the connective properties of regions previously implicated in the identification of emotional stimuli and ascription of value and motivation (as previously ascribed to the functional properties of the mesolimbic DA pathway; Haber & Knutson, 2010). We further sought to map variability in PA. Our interest in positive emotionality here follows from prior evidence suggesting that the neurofunctional properties of subcortical units within the mesolimbic dopamine pathway may influence expressions of positive emotions and motivated behavior. Prior work has convincingly linked heterogeneity in the expression of negative affectivity to neural connectivity principles among individuals with high anxiety (Evans et al., 2020; Mizzi et al., 2022; Price et al., 2020). However these same principles have not yet been extended to understanding heterogeneity within the expression of *positive affectivity* in this population. As such, the current study sought to characterize functional connectivity differences in the reward pathway that may contribute to the heterogeneity in observations of PA and motivational deficits in individuals with SAD. Prior functional connectivity evidence has probed similar questions in SAD, although not explicitly related to the relationship between PA and reward, and has found increased functional connectivity between regions associated with reward and emotional processing such as the VS, the dorsal ACC (dACC), and the vmPFC, as well as other regions such as frontoparietal and subgenual cingulate regions (Anteraper et al., 2014; Manning et al., 2015). Therefore, given the implicated role of reward circuitry dysfunction and PA in the maintenance of SAD as described above, the present study aimed to examine functional connectivity differences in reward networks (i.e., VS, OFC, vmPFC, and ACC) and directly investigate the relation between reward connectivity and self‐reported PA to determine if functional connectivity differences between the VS and areas within the OFC, vmPFC, and ACC are also associated with diminished PA.

The empirical basis for presupposing a relationship between PA and diminished functional connectivity among reward‐related regions lies in prior work suggesting that positive affect itself may be the joint output of two constituent DA‐driven systems: “liking” (hedonic impact of events) and “wanting” (incentive salience of environmental stimuli). In turn, these constituent processes are uniquely attributable to distinctive components of mesolimbic DA signaling (Nguyen et al., 2021). For example, liking appears to be linked more strongly to basal ganglia signaling, particularly ventral striatum (VS) whereas wanting appears to involve a larger and more anatomically distributed mesolimbic dopamine circuit involved in incentive motivation to obtain and consume rewards (Morales & Berridge, 2020). Although, it should be noted neither liking nor wanting are constrained with absolute exclusivity to certain anatomical locations and indeed considerable overlap exists (Berridge et al., 2009). These core processes and their neural generators are empirically distinguishable but are thought to jointly give rise to the conscious and subjective experience of positive affect (Nguyen et al., 2021). In support of this notion, Nikolova et al. (2012) probed the relationship between evoked responses in VS and self‐reported positive affect in functional MRI data, by examining whether VS responses to positive and negative feedback during a number guessing paradigm interacted with life stress to predict self‐reported state PA. Results indicated that lower levels of life stress were associated with higher PA for participants with relatively high but not low VS reactivity to feedback. Interestingly, Qi and colleagues (2020) reported that PA was inversely related to functional connectivity estimates of salience and emotion networks, suggesting that the connective properties of regions involved in the integration of sensory, emotional, and cognitive information may also be involved in self‐reported PA, although this effect in socially anxious samples remains unknown.

Thus, the current study aimed to (1) evaluate reward functional connectivity coupling strengths in adults with SAD versus controls; (2) assess the relationship between functional connectivity strength and self‐reported PA within SAD and control groups; and (3a) establish within‐group heterogeneity of reward couplings and (3b) self‐reported PA and compare heterogeneity markers between groups. As related to the specific aims above, we hypothesized that as compared to controls, adults with SAD would demonstrate hyperconnectivity between the VS and the OFC, the vmPFC, and the ACC. Furthermore, we expected that self‐reported indices of PA would relate to functional connectivity strength such that diminished self‐report of PA would correlate with hyperconnectivity in adults with SAD between the VS and the OFC, vmPFC, and ACC as compared to controls. Additionally, as related to heterogeneity in reward presentations we predicted that controls would have less variability within connectivity strengths as compared to the SAD group. Lastly, we hypothesized that individuals with SAD would demonstrate more heterogeneity in reward ROI‐ROI connectivity and self‐reported PA expression relative to controls.

## 2 METHODS AND MATERIALS

### 2.1 Participants

Thirty‐three healthy controls and thirty‐one participants who met diagnostic criteria for SAD were included in the present study. This study was reviewed and approved by the local Institutional Review Board (IRB; #20‐346) at Virginia Tech and written informed consent was obtained from all participants. Participants included individuals from a previous study (Richey et al., 2017) as well as additional participants from separate imaging trials. Inclusion criteria for all participants included a primary diagnosis of SAD, or no present clinical diagnoses for the control group, however past presence of diagnosis was allowed. Participants were excluded if they had a history of epilepsy, head trauma, implanted metal in their body, or other restrictions barring examination via MRI (e.g., pacemakers, cochlear implants, aneurysm clips, etc.). The average age of participants was 24.8 years (*SD* = 6.9). The majority of the sample identified as cisgender men (55%), with the remaining sample identifying as cisgender women (43.3%) or as a transgender woman (1.7%). The racial and ethnic identity breakdown of the final sample is as follows: White (78.3%), Asian (16.7%), American Indian/Alaskan Native (1.5%), and Native Hawaiian/Other Pacific Islander (1.5%). Approximately 25.8% of participants in the SAD group reported a previous psychological diagnosis including generalized anxiety disorder (12.9%), a specific phobia (6.5%), and depression (3.2%), and obsessive‐compulsive disorder (3.2%). For the control group, approximately 6% of participants in the SAD group reported a previous psychological diagnosis including generalized anxiety disorder (3%) and depression (3%). Additionally, approximately 7% of the participants also reported receiving some form of treatment for psychological issues in the past, and no participants were currently enrolled in any psychological treatment during the present study.

### 2.2 Procedures

All participants were administered the Anxiety Disorders Interview Schedule—IV (ADIS‐IV; Grisham et al., 2004) by a research reliable graduate student in order to determine the presence or absence of SAD. For participants in the SAD group, SAD was required to be the principal diagnosis, although other comorbid anxiety disorders were allowed. If other conditions were present, the determination of the principal diagnosis was made according to the highest clinical severity ratings (CSR). The control sample included only individuals that did not meet a current clinical threshold for any psychological disorder. Additional exclusion criteria for participants have been reported previously (Richey et al., 2017).

### 2.3 Measures

#### 2.3.1 Anxiety Disorders Interview Schedule‐IV (Grisham et al., 2004)

The ADIS‐IV is a semistructured interview that assesses the presence of psychiatric disorders according to DSM‐IV criteria. Note that some data for this study were collected prior to the publication of the ADIS‐5, and we opted to retain the initial interview assessment instrument to ensure comparability across participants in screening procedures. This assessment permits differential diagnosis between anxiety disorders and other disorders and provides a CSR severity rating ranging from 0 (“no disorder present”) to 8 (“patient is in need of hospitalization”). Interrater reliability was high (kappa = .80 or above).

#### 2.3.2 Liebowitz Social Anxiety Scale (LSAS; Liebowitz, 1987)

The LSAS is a 24‐item questionnaire that assesses social anxiety symptomatology. The LSAS features subscales for both fear and avoidance of social interactions and performance situations and has good psychometric properties (LSAS total α = .97). The LSAS total score was used to characterize the groups, and to confirm that groups differed in self‐reported social anxiety.

#### 2.3.3 Positive and Negative Affect Schedule (PANAS; Watson et al., 1988)

The PANAS is a self‐report questionnaire that measures both positive and negative affect. Participants were asked to indicate the extent to which they have felt a certain way (e.g., alert, attentive, nervous, upset) over the course of the past week when they completed the neuroimaging scan. The PA and NA scales of the PANAS were used as variables for brain‐to‐behavior correlations. In the current study, the PANAS showed good internal consistency (PA α = .81; NA α = .76).

### 2.4 Data analysis

#### 2.4.1 Neuroimaging data acquisition

Resting‐state neuroimaging data were collected on a Siemens TrioTim 3T scanner system with 50‐mT/m gradients (Siemens, Erlangen, Germany). Head movement during scanning was minimized through the use of foam cushions. High‐resolution T1‐weighted images were collected using a rapid gradient echo sequence (TR/TE: 2600 ms/3.02 ms; FOV: 22 cm; Image matrix: 184 × 256 × 192; voxel size = 0.82 mm × 1.00 mm × 1.00 mm). Functional imaging used an EPI sequence with a repetition time (TR) of 2000 ms, echo time (TE) = 25 ms, flip angle = 90°, 220 mm field of view (FOV), 64 × 64 matrix. Functional slices were oriented 30° superior‐caudal to the plane through the anterior and posterior commissures (Deichmann et al., 2003). Each functional image was acquired in an interleaved format, comprising 37.4 mm axial slices for measurement of the BOLD effect, yielding 3.4 mm × 3.4 mm × 4.0 mm voxels. Runs began with 5 discarded RF excitations to allow for steady state equilibrium.

#### 2.4.2 fMRI preprocessing

Raw resting‐state imaging data was pre‐processed using tools from the Configurable Pipeline for the Analysis of Connectomes (C‐PAC; Craddock et al., 2013).^1^ C‐PAC builds upon pre‐existing fMRI analysis software packages to create data analytic pipelines. Furthermore, C‐PAC was used for slice time correction, and spatial smoothing, and high‐pass temporal filtering. Motion and noise sources were “denoized” and regressed out accordingly using C‐PAC's “Nuisance Corrections” tools and visually inspected for other noise or artifactual components.

#### 2.4.3 Regions of interest (ROIs)

**FIGURE 1:**
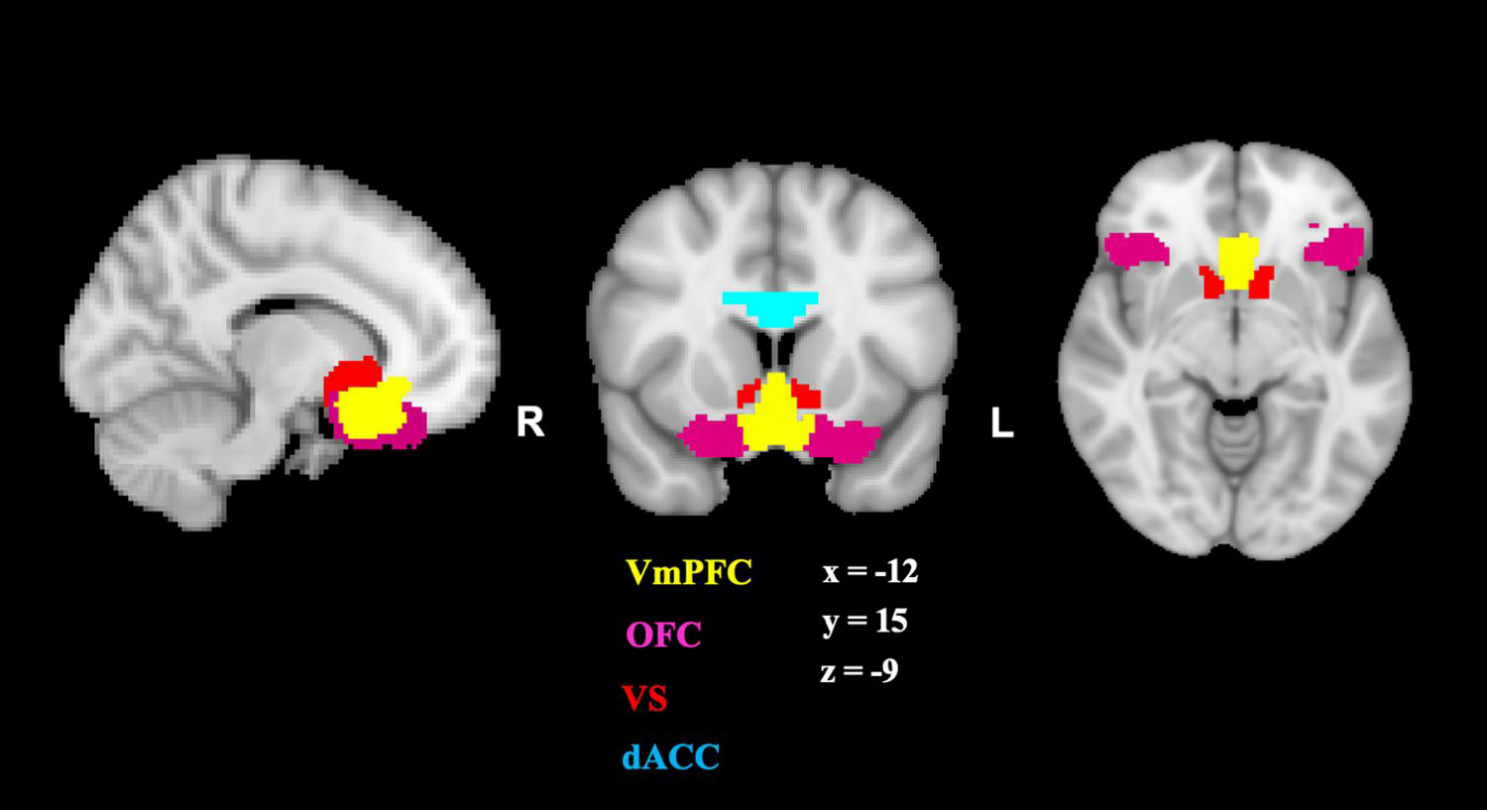
Reward regions of interest (ROIs) in the present study. Note: dACC here represents the subgenual and cingulate masks.

ROIs were chosen to evaluate functional connectivity in reward regions based on their functional roles in reward processing. ROIs were defined using the Craddock 200 parcellation atlas (Craddock et al., 2012). The Craddock atlas specifies ROIs into spatially coherent regions of homogenous functional connectivity and was derived from resting‐state fMRI data rather than anatomical or cyto‐architectonic boundaries. Specifically, as reviewed in the introduction, important nodes of influence for the reward network include the VS, OFC, vmPFC, and the ACC (as indexed by the cingulate and subgenual in the present study; see Figure 1). An ROI‐based connectivity analysis was used to assess connectivity strength between ROIs for each group. This was accomplished by extracting the time series from each ROI with C‐PAC, such that the mean of each ROI across all time points was extracted and then correlated with ROIs. Additionally, other brain regions (i.e., bilateral insula and bilateral occipital cortex [OC]) were included as control regions. Bilateral insula and OC were chosen as control regions in the present study as each region represents non‐dopaminergic control regions that have not been shown to be associated with PA deficits. Table 1 below displays MNI coordinates for all ROIs and control regions. Next, ROI‐based timeseries correlations were carried out using NiTime (http://nipy.sourceforge.net/nitime) among ROI couplings such that the correlation between each ROI‐to‐ROI coupling was extracted. Code for this analysis can be found at https://github.com/corinnecarlton/RSP.git.

**TABLE 1:** MNI coordinates for ROIs and control regions

***ROIs***	***X***	***Y***	***Z***
R VS	+10	+12	–7
L VS	–10	+12	–7
R vmPFC	+9	+42	–17
L vmPFC	–9	+42	–17
R OFC	+28	+25	–12
L OFC	–28	+25	–12
R Cingulate	+11	+25	+20
L Cingulate	–11	+25	+20
R Subgenual	+10	+24	–13
L Subgenual	–10	+24	–13
***Control regions***			
R Insula	+58	–25	+1
L Insula	–58	–25	+1
R OC	+20	–96	+3
L OC	–20	–96	+3

L = left; R = right; VS = ventral striatum; vmPFC = ventromedial prefrontal cortex; OFC = orbitofrontal cortex; OC = occipital cortex.

#### 2.4.4 Statistics

Means for self‐report and demographic information by group are presented in Table 2. Differences in participant characteristics across groups were examined using independent samples *t*‐tests. Sex ratios and race were also examined using chi‐square tests. Data were first screened for relevant statistical assumptions: all measures were found to be normally distributed, and data were collected independently; however, since groups had statistically significantly difference variances with regard to connectivity, a Welch's *t*‐test were applied to ROI‐ROI coupling analyses. Thus, Welch's *t*‐tests were run to assess group mean differences in behavioral variables and in order to test aim 1 and determine whether groups (SAD vs. controls) differed in ROI‐to‐ROI connectivity strength. Secondarily, in order to test aim 2 and examine brain and behavior relationships between connectivity strength and PA, significant group differences resulting from ROI‐to‐ROI functional connectivity were correlated with self‐reported PA measures, within groups. Because the direct interpretation of manifold pairwise comparisons (9 + 8 + 7 + 6 + 5 + 4 + 3 + 2 + 1, in our case) has the clear potential to elevate Type I error, we implemented a false discovery rate (FDR) correction (0.05 in our case), as described in detail by Benjamini and Hochberg (1995), and implemented here using the python module *statsmodels*. As such, the *p* values reported here have been previously adjusted for multiple hypothesis testing to limit FDR. Additionally, exploratory brain‐behavior correlations were run for all ROI‐ROI connections to examine associations by group. In order to test aim 3a and examine the heterogeneity of functional connectivity strength, we examined the variability (as represented by standard deviation) within each ROI coupling within groups, and compared between‐group variability in connectivity strengths via independent *t*‐test, such that a smaller amount of variance within a group indicated less heterogeneity than those with a larger amount of variability. To test aim 3b, we examined the variance of PA and NA by computing within‐group intraclass correlation coefficients (ICC) for PA and NA and comparing amongst groups using an independent samples *t‐*test.

**TABLE 2:** Means and *SD* for demographics and social anxiety and affect behavioral measures by group

	**SAD (*N* = 31)**	**Control (*N* = 33)**	***p* Value**
**Gender (% cisgender woman)**	32%	38%	.40
**Race**			.55
**White**	82%	79%	
**Asian**	14%	17%	
**American Indian/Alaskan Native**	4%	‐	
**Native Hawaiian/Pacific Islander**	‐	4%	

	**Mean**	***SD***	**Mean**	***SD***	***p* Value**
**Age**	25.8	7.6	24.2	6.5	.30
**LSAS Total**	69.41	18.43	19.03	10.50	<.001
**PANAS PA**	28.11	5.93	30.56	9.41	.25
**PANAS NA**	15.77	4.33	12.22	4.27	<.01

LSAS = Leibowitz Social Anxiety Scale; PANAS = Positive and Negative Affect Schedule; PA = positive affect; NA = negative affect.

## 3 RESULTS

### 3.1 Group characteristics

Independent samples *t*‐tests were run in order to assess for group differences in age. There were no statistically significant differences between‐group means for age (*p* = .30). Furthermore, a chi‐square analysis was run to examine race and gender ratios across groups. Results indicated no significant differences in race across groups (*X* ^2^ (3, *N*  = 64) = 2.09, *p*  = .55), nor gender differences across groups (*X* ^2^ (2, *N*  = 64) = 1.83, *p*  = .40); nor with regard to gender status (*X* ^2^ (1, *N*  = 64) = 1.18, *p*  = .21). Independent samples *t*‐tests were also run to examine differences in behavioral variables across groups. The LSAS total mean was significantly higher in the SAD group as compared to controls, as expected (*p* < .001). Mean PA scores were demonstrated to be lower for the SAD group, though not significantly different from controls (*p* = .25) and NA scores were significantly higher in the SAD group versus controls (*p* < .01).

### 3.2 Functional connectivity

Average correlational values for ROI‐to‐ROI couplings by group are displayed in Table 3. Results from Welch's *t‐*test analyses revealed significant differences between‐group means for ROI couplings between the left OFC and right OFC (*t* (1, 62) = 4.07, *p* < .05), with the SAD group demonstrating weaker connectivity between areas (*r* = .80) than controls (*r* = .86). Additionally, trend‐level significance emerged between groups for the ROI coupling between the right VS and right insula (*t* (1, 62) = 2.96, *p* = .09) such that the SAD group exhibited weaker connectivity between areas (*r* = .42) as compared to controls (*r* = .50). However, no significant differences among the VS and vmPFC nor the VS and the OFC emerged. Additionally, significant findings emerged between‐group means for the right VS and the right OC and left OC areas (*t* (1, 62) = 11.09, *p* < .05; *t* (1, 62) = 10.36, *p* < .05, respectively) such that the SAD group demonstrated weaker connectivity between areas (*r* = .34 to .36) as compared to controls (*r* = .53 to .55).

**TABLE 3:** Average connectivity strengths between ROIs

	1	2	3	4	5	6	7	8	9	10	11	12	13	14
*SAD*														
1. L VS	‐													
2. R VS	.75	‐												
3. L vmPFC	.43	.38	‐											
4. R vmPFC	.39	.40	.86	‐										
5. L OFC	.58	.53	.49	.41	‐									
6. R OFC	.57	.56	.50	.52	.80*	‐								
7. L Insula	.46	.43	.27	.22	.78	.69	‐							
8. R Insula	.46	.42	.29	.27	.67	.76	.88	‐						
9. L Cingulate	.67	.64	.63	.67	.67	.67	.52	.50	‐					
10. R Cingulate	.59	.68	.63	.64	.62	.71	.47	.48	.91	‐				
11. L Subgenual	.55	.52	.47	.41	.69	.66	.77	.74	.64	.58	‐			
12. R Subgenual	.52	.53	.42	.42	.67	.68	.76	.75	.62	.58	.97	‐		
13. L OC	.40	.34**	.35	.28	.52	.43	.45	.43	.30	.27	.46	.45	‐	
14. R OC	.41	.36**	.35	.31	.52	.47	.44	.46	.31	.29	.46	.46	.91	‐
*Control*														
1. L VS	‐													
2. R VS	.77	‐												
3. L vmPFC	.41	.38	‐											
4. R vmPFC	.36	.37	.87	‐										
5. L OFC	.58	.52	.56	.53	‐									
6. R OFC	.57	.56	.51	.52	.86*	‐								
7. L Insula	.46	.47	.30	.28	.76	.72	‐							
8. R Insula	.50	.50	.28	.26	.70	.78	.90	‐						
9. L Cingulate	.66	.58	.65	.63	.69	.69	.50	.52	‐					
10. R Cingulate	.58	.61	.63	.67	.67	.70	.50	.51	.93	‐				
11. L Subgenual	.53	.56	.46	.43	.70	.68	.77	.74	.64	.61	‐			
12. R Subgenual	.54	.58	.43	.45	.69	.70	.80	.80	.62	.63	.97	‐		
13. L OC	.50	.53**	.33	.28	.49	.46	.48	.49	.31	.29	.47	.48	‐	
14. R OC	.50	.55**	.33	.29	.50	.47	.49	.51	.31	.31	.49	.51	.92	‐

*Note*: Significantly different connectivity strengths between SAD and controls are bolded and include an asterisk (*) for *p* < .05, two asterisks (**) indicate significant differences at *p* < .01, and items bolded alone indicate trend‐level significance (*p* = .08).

L = left; R = right; VS = ventral striatum; vmPFC = ventromedial prefrontal cortex; OFC = orbitofrontal cortex; OC = occipital cortex.

### 3.3 Brain‐behavior relations

Correlational analyses were performed to examine brain‐behavior relationships of both groups. As such, the relationships between ROI couplings and PA were examined through correlational analyses by group. Results for brain‐behavior analyses are displayed in Table 4. Results demonstrated significant negative relationships in the SAD group between PA and the ROI couplings of right OFC and left insula (*r* = –.38, *p* < .05), right OFC and right insula (*r* = –.45, *p* < .05), right OFC and left subgenual cingulate (*r* = –.44, *p* <.05), right OFC and right subgenual cingulate (*r* = –.41, *p* < .05), left insula and left cingulate (*r* = –.41, *p* <.01), left insula and right cingulate (*r* = –.50, *p* <.05), right insula and right cingulate (*r* = –.45, *p* <.05), left cingulate and right subgenual cingulate (*r* = –.38, *p* <.05), right cingulate and left subgenual cingulate (*r* = –.59, *p* <.01), and right cingulate and right subgenual cingulate (*r* = –.54, *p* <.01). No significant relationships were revealed for PA and ROI couplings within the control group.

**TABLE 4:** Correlation values between connectivity strengths, PA, and NA

	**1**	**2**	**3**	**4**	**5**	**6**	**7**	**8**	**9**	**10**	**11**	**12**	**13**	**14**
***PA***														
1. L VS	‐	.04	−.20	−.09	−.14	−.11	−.03	.01	−30	−34	.09	.15	.03	−.04
2. R VS	.20	‐	−.04	.08	−.02	−.13	−.08	−.18	−.26	−.34	−.15	−.09	.30	.19
3. L vmPFC	−.12	−.06	−	.15	−.15	.05	.09	.10	−.25	−.16	.19	.16	.05	.04
4. R vmPFC	−.06	−.01	.14	‐	.13	.21	.21	.25	−.21	−.15	.28	.28	.13	.09
5. L OFC	.16	.06	.17	.10	‐	.07	.21	.03	−.24	−.12	.15	.21	−.01	−.06
6. R OFC	−.06	−.02	.00	.04	−.08	‐	.14	.17	−.06	−.15	.19	.26	.06	−.03
7. L Insula	.16	.07	.00	−.07	−.14	−.38*	‐	−.16	.12	.14	.08	.11	−.05	−.14
8. R Insula	.05	.00	−.07	−.11	−.06	−.45*	−.05	‐	.26	.19	.07	.20	−.05	−.13
9. L Cingulate	−.09	−.25	.13	.17	−.29	−.18	−.41**	−.28	‐	.12	.33	.28	−.26	−.29
10. R Cingulate	−.20	−.25	.05	.15	−.21	−.18	−.50*	−.45*	−.02	‐	.33	.26	−.23	−.22
11. L Subgenual	.06	.04	−.21	−.28	−.05	−.44*	−.04	−.23	−.37	−.59**	‐	−.13	−.05	−.12
12. R Subgenual	.05	.02	−.13	−.21	−.13	−.41*	−.10	−.30	−.38*	−.57**	−.07	‐	.04	−.03
13. L OC	.18	.21	.00	.03	.07	−.12	.25	.17	−.01	–.15	.26	.24	‐	−.09
14. R OC	.18	.18	−.03	−.04	.02	−.22	.22	.13	−.07	−.22	.23	.19	.13	−
***NA***														
1. L VS	‐	−.22	−.10	−.10	.05	.17	.11	.23	−.23	−.16	.04	.06	−.11	−.10
2. R VS	.47**	‐	−.01	.07	−.02	.01	.04	.02	−.11	−.06	−.05	−.03	−.09	−.12
3. L vmPFC	−.03	.11	‐	.00	−.01	−.10	.07	.06	.22	.10	.09	.05	−.12	−.10
4. R vmPFC	−.07	.02	−.31	‐	−.18	−.18	.04	.02	.04	−.06	.08	.02	−.07	−.04
5. L OFC	.17	−.01	.16	.13	‐	.09	.10	.29	−.11	−.06	.11	.13	−.07	.00
6. R OFC	.27	.06	.25	.07	.26	‐	.04	.19	−.20	−10	.11	.15	−.08	−.04
7. L Insula	.20	.06	.11	.14	−.16	.18	‐	.01	−.01	.06	.27	.25	.10	.15
8. R Insula	.30	.23	.20	.15	.13	.15	.16	‐	−.02	.09	.19	.24	.00	.04
9. L Cingulate	.20	−.06	.33	.26	.18	.28	.11	.32	‐	−.14	.01	−.08	−.24	−.17
10. R Cingulate	.13	−.21	.37	.20	.22	.17	.19	.30	.34	‐	.18	.13	−.14	−.09
11. L Subgenual	.18	.21	.08	.20	−.12	.07	.02	.14	.15	.19	‐	.06	.10	.13
12. R Subgenual	.25	.17	.06	.17	−.16	−.02	.05	.03	.05	.10	.24	‐	.08	.12
13. L OC	−.04	.07	−.28	−.27	.04	−.11	−.10	−.15	−.04	−.07	−.27	−.26	‐	−.03
14. R OC	.07	.37	−.13	−.20	.02	−.12	−.11	−.17	.04	.02	−.24	−.30	.14	‐

*Note*: Values falling underneath the diagonal represent the SAD group; values above the diagonal represent the control group. Significantly correlated brain‐behavior relationships are bolded and include an asterisk (*) for *p* < .05; two asterisks (**) indicate significant differences at *p* < .01.).

L = left; R = right; VS = ventral striatum; vmPFC = ventromedial prefrontal cortex; OFC = orbitofrontal cortex; OC = occipital cortex.

Brain‐behavior correlations were run for NA as well, with results showing that the SAD group demonstrated a significant positive correlation between NA and the left VS to right VS ROI coupling (*r* = .47, *p* <.01). No significant relationships were revealed for NA and ROI couplings within the control group.

### 3.4 Heterogeneity of functional connectivity

The variance within each ROI coupling was examined within groups, and compared between groups in terms of magnitude in order to assess heterogeneity among connectivity strength estimates in reward presentation between the SAD and control groups. Results of *t*‐tests indicated that the SAD group exhibited more significantly more (*p*s < 0.05) variability across many of the ROI couplings, including VS, vmPFC, and OFC reward couplings as compared to controls (see Table 5).

**TABLE 5:** Variance values within connectivity strengths for ROI couplings

	**1**	**2**	**3**	**4**	**5**	**6**	**7**	**8**	**9**	**10**	**11**	**12**	**13**	**14**
***SAD***														
1. L VS	‐													
2. R VS	**.05**	‐												
3. L vmPFC	**.11**	**.13**	‐											
4. R vmPFC	**.14**	**.14**	.01	‐										
5. L OFC	**.08**	**.07**	**.12**	**.15**	‐									
6. R OFC	.05	.05	**.06**	**.08**	**.02**	‐								
7. L Insula	**.07**	**.06**	**.09**	**.12**	.01	.03	‐							
8. R Insula	**.05**	**.06**	**.07**	.08	**.04**	.02	**.02**	‐						
9. L Cingulate	**.08**	.05	**.10**	**.10**	.03	**.05**	**.05**	**.05**	‐					
10. R Cingulate	**.06**	.06	**.06**	**.05**	**.05**	**.03**	**.06**	**.05**	**.01**	‐				
11. L Subgenual	.04	**.05**	**.06**	**.08**	.02	.04	.01	**.02**	**.03**	**.04**	‐			
12. R Subgenual	**.04**	**.05**	**.06**	**.09**	.02	**.04**	.01	**.02**	**.02**	**.03**	.00	‐		
13. L OC	**.08**	**.08**	.07	**.09**	.07	.08	.07	.06	**.08**	**.09**	.07	.06	‐	
14. R OC	**.07**	**.07**	.07	.08	.05	.05	.04	.03	.07	.08	.03	.03	.00	‐
***Control***														
1. L VS	‐													
2. R VS	.01	‐												
3. L vmPFC	.07	.06	‐											
4. R vmPFC	.07	.07	.01	‐										
5. L OFC	.05	.06	.04	.05	‐									
6. R OFC	.05	**.06**	.05	.07	.01	‐								
7. L Insula	.06	.05	.07	.08	.01	.03	‐							
8. R Insula	.03	.03	.06	.08	.01	.02	.01	‐						
9. L Cingulate	.03	**.06**	.03	.02	.03	.02	.04	.02	‐					
10. R Cingulate	.04	.06	.03	.02	.04	.02	.04	.02	.00	‐				
11. L Subgenual	.04	.03	.05	.04	**.03**	.04	.01	.01	.01	.02	‐			
12. R Subgenual	.02	.03	.05	.04	**.03**	.03	.01	.01	.01	.02	.00	‐		
13. L OC	.04	.04	**.08**	.07	**.08**	.08	**.10**	**.09**	.07	.08	**.08**	**.07**	‐	
14. R OC	.05	.04	**.09**	.08	**.08**	**.08**	**.09**	**.07**	.07	.08	**.07**	**.07**	**.01**	‐

*Note*: Variance scores that are significantly higher for one group as compared to the other group are bolded (*p* <.05).

L = left; R = right; VS = ventral striatum; vmPFC = ventromedial prefrontal cortex; OFC = orbitofrontal cortex; OC = occipital cortex.

### 3.5 Heterogeneity of affective presentation

With regard to behavioral measures of affect, the intraclass correlation coefficient (ICC) was computed for PA and NA within groups, and then compared between groups. Results indicate that the SAD group demonstrated a weaker ICC for both PA (ICC = .77) and NA (ICC = .77) than controls (PA ICC = .93; NA ICC = .90), suggesting that on a descriptive level via the ICC, the SAD group may have been more heterogeneous in its affective presentation. However, while the SAD group demonstrated lower mean level PA and a lower ICC for PA, these values are not statistically significantly different as compared to controls (*p* = .25).

## 4 DISCUSSION

The purpose of the present study was to investigate functional connectivity of reward areas and links to heterogeneity of PA in individuals with SAD versus controls. Given prior work that has indicated that individuals with SAD exhibit diminished reward circuitry activation during social reward processing (Becker et al., 2017; Cremers et al., 2015; Richey et al., 2017) and other work indicating hyperconnectivity between reward regions and emotional processing regions in individuals with SAD (e.g., Anteraper et al., 2014), we hypothesized that individuals with SAD would demonstrate altered functional connectivity between the VS and OFC, vmPFC, and ACC (indexed by the cingulate and subgenual cingulate in the present study). Contrary to our hypothesis regarding hyperconnectivity of reward ROIs in individuals with SAD, results from ROI‐based analyses of resting‐state data indicated that the SAD group demonstrated significantly diminished connectivity strengths between ROI couplings, as compared to controls. Additionally, the SAD group demonstrated trend level diminished connectivity strength between right VS and right insula. These results are somewhat in line with previous reports (e.g., Manning et al., 2015), which found decreased functional connectivity between the VS and other regions of reward. Moreover, the present results support the idea that SAD seems to be associated with widespread differences in functional connectivity strengths within the reward system (e.g., Manning et al., 2015). Results from the present study demonstrated significantly weaker associations among the right VS and right OC as well as right VS and left OC in individuals with SAD as compared to controls. This was unexpected as we chose the OC as a control region give its non‐dopaminergic input. However, at least one prior study has demonstrated weaker connectivity in frontal lobe to OC in individuals with SAD as compared to controls (e.g., Ding et al., 2011). Thus, connectivity among reward regions and typical control regions, such as the OC, should be further investigated in future studies.

In light of previous studies that have established low PA as a feature of SAD (Alden et al., 2008; Brown et al., 1990; Kashdan, 2007; Watson et al., 1988), as well as work suggesting an interface between anhedonia (which comprises a diminished PA component) and altered connectivity in reward regions (e.g., Pornpattananangkul et al., 2019; Wang et al., 2016), we hypothesized that diminished self‐report of PA would relate to altered connectivity of reward regions in individuals with SAD as compared to controls. Results from the present investigation revealed a number of interesting patterns. Regarding our hypothesis, no ROI couplings that were significantly different for the SAD group versus the control group emerged as significantly correlated to PA, and thus our hypothesis was unsupported. Brain‐behavior correlations were also run for NA, with a significant positive relationship emerging within the SAD group only between NA and the ROI coupling of left VS and right VS. However, exploratory analyses of all ROI couplings were also carried out to characterize brain‐behavior relationships between ROI couplings and PA and NA. Results from these exploratory analyses demonstrated that PA was associated with aberrant reward connectivity alterations in the SAD group only. These results suggest that PA expression shows a unique pattern that relates to reward circuitry connectivity strength in individuals with SAD, despite the connectivity strengths not being significantly different from controls.

Regarding our final aim, we hypothesized that the control group would demonstrate lower variability within ROI couplings within their group as compared to the control group. This hypothesis was grounded in prior work that has shown that some, but not all individuals with SAD exhibit diminished PA (e.g., Nelemans et al., 2014), and by extension, if diminished PA was indeed an output of diminished crosstalk between reward regions, then not all individuals with SAD would exhibit altered reward circuitry. Results from the present investigation indicate that our hypothesis was partially supported. Through examining the relative variance each group demonstrated within ROI couplings, it was determined that the SAD group showed more variability within reward‐to‐reward ROI couplings, as indicated by larger variance values than the control group. Of note, the control group seemed to exhibit more variance in reward‐to‐control areas as compared to the SAD group. Additionally, group heterogeneity differences in PA and NA were examined. On the surface level, results indicated that individuals within the SAD group had greater heterogeneity in their reporting of PA as compared to controls. This is consistent with prior work indicating that PA in SAD is variable, and that only some individuals with SAD may demonstrate this deficit. However, these findings should be considered in light of no statistically significant differences in PA demonstration among individuals with SAD as compared to controls in the present sample. These distinctions are important for considering future treatment approaches for SAD, such that the assessment of PA before treatment may be informative for clinicians when considering appropriate treatment approaches for symptom presentation (e.g., Carlton et al., 2021; Carlton et al., 2021; Richey et al., 2019; Strege et al., 2018); thus, further work intentionally recruiting samples of adults with SAD with co‐occurring diminished PA is warranted.

The results from the present study may also hold significance for the development of interventions for SAD. Specifically, the present study revealed select instances of hypoconnectivity between reward regions in individuals with SAD, and also revealed specific reward ROI couplings associated with diminished PA. These findings may be informative for the creation and modification of intervention approaches for individuals with SAD that focus on enhancing PA in an attempt to heighten reward responsivity to social rewards. Of interest here, recent research points to promising evidence that improvements in PA relate to significant improvements in SAD symptoms (Strege et al., 2018). For example, a study by Taylor and colleagues (2017) demonstrated that increases in PA was the most robust predictor for increases in connectedness as measured by a relationship‐building question (i.e., “how connected to your partner do you feel?”) as compared to reduction of anxiety symptoms in a sample of individuals with SAD. Taken together, the results from the present study add to the literature suggesting that PA for individuals with SAD may be intimately linked with disruptions of reward processing. As this work was primarily focused on reward connectivity broadly, and SAD may be specifically impacted by social anhedonia (see Richey et al., 2019 for review), it will be important to examine reward connectivity using socially based tasks, as this may aid in the specificity of mechanistic findings for treatment development.

As with any study, a number of limitations should be noted. Despite being able to examine ROI couplings in conjunction with behavioral measures, we only had access to the 20‐item PANAS, thus limiting our analyses to broad domains of PA and NA. The utilization of extended versions of the PANAS would have allowed us to examine facets of PA that may be more specifically related to individuals with SAD and resulting reward regions, such as motivationally valenced facets of PA (Carlton et al., 2021). Therefore, future work regarding PA and SAD should use the extended PANAS to probe motivationally valenced facets of PA and the relationship of those with reward functional connectivity in individuals with SAD. Additionally, the current study used an ROI‐to‐ROI‐coupling‐based approach, which allows an assessment of connectivity strength between ROI couplings, but not directionality. Therefore, future work should examine the directionality of relationships, perhaps using the Granger causality. Lastly, our sample consisted of a majority of White/Caucasian men. As such, findings here should be considered in light of their ability to generalize and should be reconfirmed in more diverse samples.

## 5 CONCLUSION

In conclusion, results from the present study identified several ROI coupling hypoconnectivity differences between SAD and control groups. Additionally, individuals with SAD displayed specific associations between reward ROI couplings and diminished PA. Lastly, the current study demonstrated that individuals with SAD had higher variability in their reward ROI coupling connectivity strength estimates and within their reports of PA as compared to controls. Results from this study provide emergent evidence for the linkage between reward regions in the brain and diminished PA in individuals with SAD, thereby reinforcing the idea that interventions for SAD should be modified to focus on enhancing PA in an attempt to increase reward responsivity to social rewards.

## FUNDING STATEMENT

This study did not receive funding.

## CONFLICT OF INTEREST STATEMENT

John Richey provides statistical consulting for Behavior LLC, unrelated to the current project. Behavior LLC has no interests financial or otherwise in the results presented herein. All other authors have no disclosures to report.

## PATIENT CONSENT STATEMENT

All participants provided informed consent.

### PEER REVIEW

The peer review history for this article is available at https://publons.com/publon/10.1002/brb3.3006.

## Supporting information


**Figure S1**. C‐PAC generic workflow for generation of motion and power statistics from online C‐PAC documentation.
**Figure S2**. C‐PAC generic workflow for extracting seed‐based data from online C‐PAC documentation.

## Data Availability

Data are available by request.

## Footnotes

1 A general diagram of C‐PAC's workflow is included in the supplementary materials (see Figures S1 and S2 in the Supplementary Materials section).
