# Supplementary material for: Associations between resting‐state neural connectivity and positive affect in social anxiety disorder
Source: Brain Behav. 2023 Apr 16;13(6):e3006. doi: 10.1002/brb3.3006 (PMC10275543; doi:10.1002/brb3.3006)
Supplement: Supplementary file 1 — Figure S1. C‐PAC generic workflow for generation of motion and power statistics from online C‐PAC documentation. Figure S2. C‐PAC generic workflow for extracting seed‐based data from online C‐PAC documentation. [file BRB3-13-e3006-s001.docx]

**Supplementary Materials.**

**
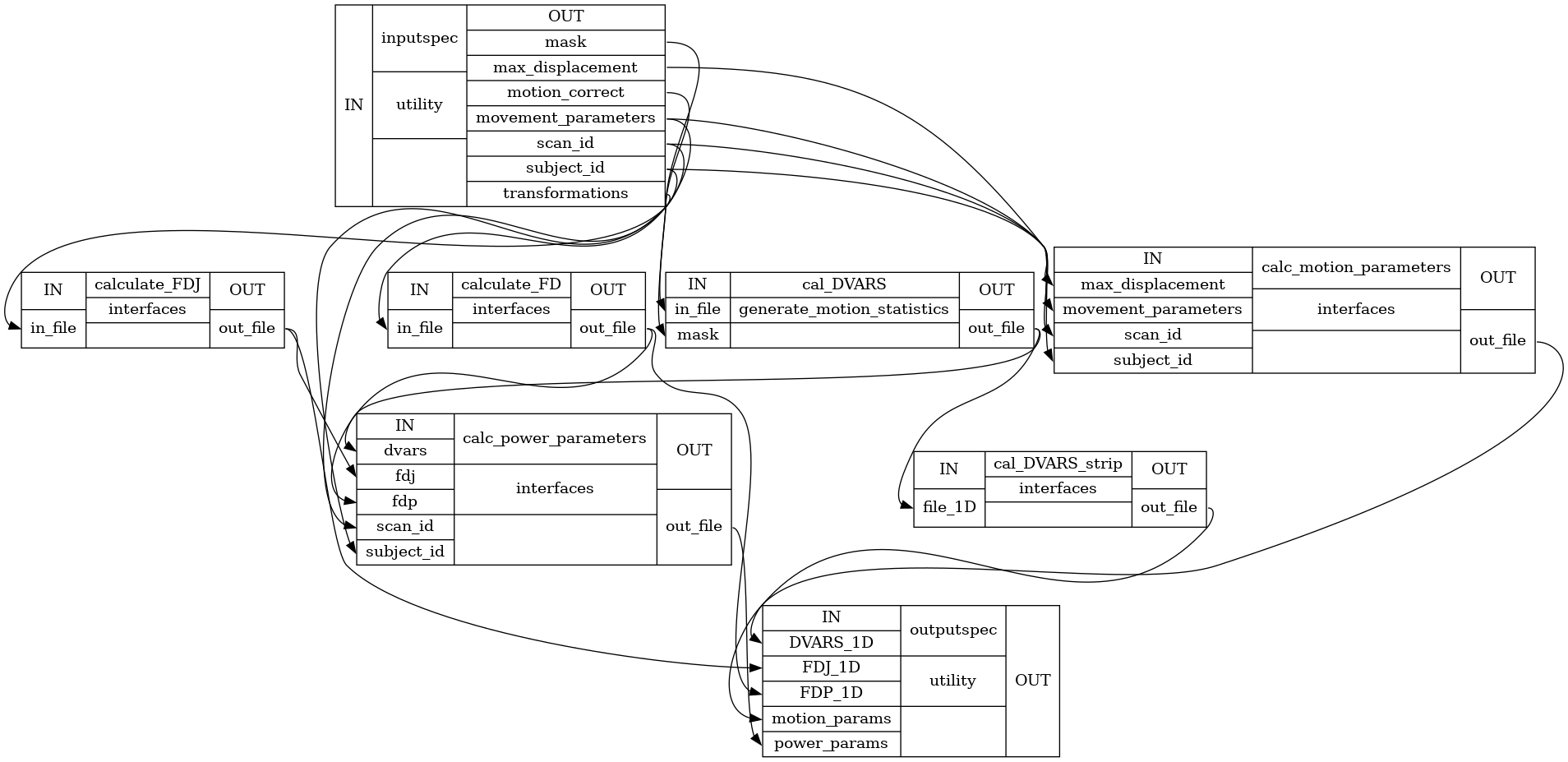
**

**Figure S1.** C-PAC generic workflow for generation of motion and power statistics from online C-PAC documentation.

**
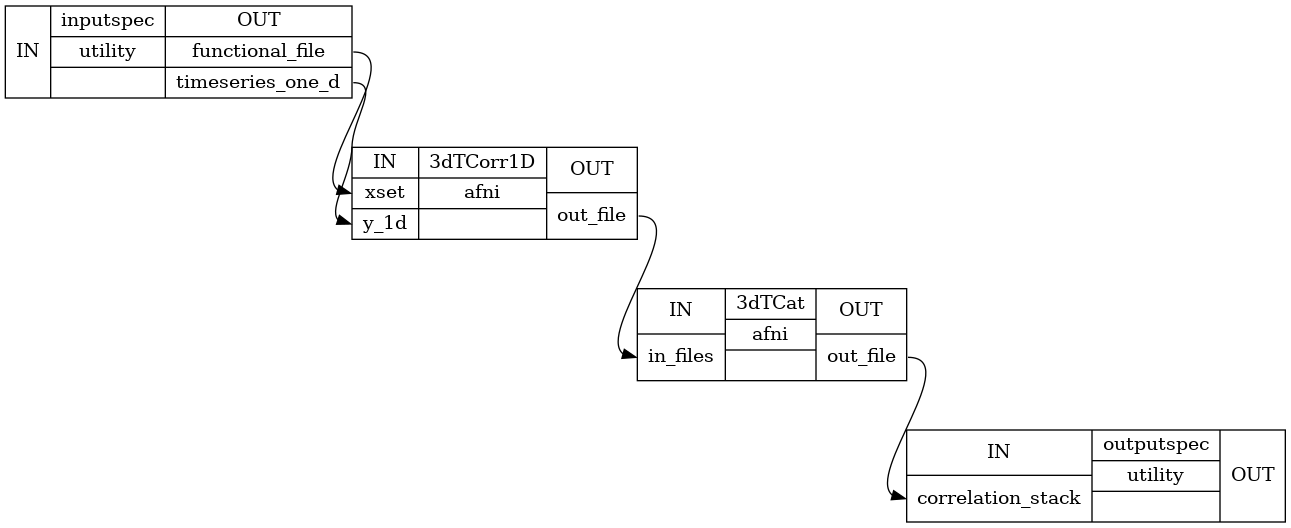
**

**Figure S2.** C-PAC generic workflow for extracting seed-based data from online C-PAC documentation.
